# Corneal Anterior and Posterior Changes in a Patient With Keratitis Associated With *Mycobacterium chelonae*: A Case Report

**DOI:** 10.1155/crop/9161714

**Published:** 2026-07-10

**Authors:** Ryo Kato, Takashi Ono, Takashi Suzuki, Yukako Taketani, Mikiko Kimakura, Tetsuya Toyono, Makoto Aihara, Takashi Miyai

**Affiliations:** ^1^ Department of Ophthalmology, University of Tokyo Hospital, Bunkyo, Tokyo, Japan, u-tokyo.ac.jp; ^2^ Department of Ophthalmology, Toho University, Ota, Tokyo, Japan, toho-u.ac.jp; ^3^ Department of Ophthalmology, Ishizuchi Eye Clinic, Niihama, Ehime, Japan

**Keywords:** contact lens, infectious keratitis, *Mycobacterium chelonae*, nontuberculous mycobacteria, ocular infection

## Abstract

**Purpose:**

This report describes a patient with anterior and posterior corneal topographic changes who developed refractory keratitis due to *Mycobacterium chelonae*, associated with soft contact lens use.

**Case:**

A 42‐year‐old man presented with blurred vision and pain in his right eye. He used disposable soft contact lenses for 2 weeks with appropriate care. He had open‐angle glaucoma, atopic dermatitis, and diabetes mellitus. At the initial presentation, a corneal epithelial defect, cell infiltration, strong hyperemia, hypopyon, and inflammation in the anterior chamber were observed. Anterior segment optical coherence tomography disclosed corneal edema and cloudiness, and the central corneal thickness had increased to 988 *μ*m. The best‐corrected visual acuity was counting fingers at 10 cm. The patient did not respond to the initial treatment with ofloxacin and steroids. The culture of the scraped sample from the contact lens case grew M. chelonae, and topical tobramycin was administered, which was effective based on antimicrobial susceptibility. It took 4 months to treat the keratitis, and the best‐corrected visual acuity improved to 0.5, although anterior and posterior corneal irregularities remained.

**Conclusion:**

Nontuberculous M. chelonae should be considered in patients with treatment‐resistant keratitis, especially in contact lens users. Topographic changes are important for evaluating corneal edema and irregular astigmatism, which impair visual function after keratitis.

## 1. Introduction

*Mycobacterium chelonae* is a nontuberculous mycobacterium found in soil, dust, and river water that causes cellulitis and abscesses in the skin and soft tissues. In ophthalmology, *M. chelonae* is a rare cause of ocular surface infections, typically following ocular trauma, surgery, contact lens use, herpetic keratitis, or long‐term steroid eye drop use [1]. This infection often presents as refractory keratitis, which is challenging to diagnose and treat due to its clinical similarity to bacterial or fungal keratitis and its resistance to many conventional antimicrobial agents.

Furthermore, keratitis caused by *M. chelonae* may exhibit satellite stromal lesions and corneal melting in the primary lesion [2], which are features that are also observed in fungal keratitis. Differentiating between the two is important to distinguish keratitis because their management strategies differ. Although endothelial plaques and anterior chamber inflammation are characteristic of fungal keratitis, these signs may not be easily detected using slit‐lamp examination of the anterior segment alone due to corneal opacity, which limits visualization [3].

Anterior segment optical coherence tomography (AS‐OCT) offers an objective method to analyze clinical findings of keratitis, including anterior chamber inflammation. Corneal topographic data, obtained via AS‐OCT, can also be used to evaluate the impact of irregular astigmatism on visual acuity. Although several case reports of keratitis caused by *M. chelonae* in contact lens users have been previously published [4–7], long‐term follow‐up using AS‐OCT to evaluate anterior and posterior corneal changes has not been previously reported.

To date, no studies have described irreversible corneal shape changes caused by nontuberculous mycobacterial keratitis as assessed by corneal tomography.

This case report presents a patient who developed refractory keratitis due to *M. chelonae* associated with soft contact lens use, along with corneal shape changes observed on AS‐OCT. The case emphasizes the importance of considering atypical pathogens in treatment‐resistant keratitis and highlights how corneal topographic changes can impact visual acuity. The purpose of this report is to describe the anterior and posterior corneal changes observed using AS‐OCT in a patient with *M. chelonae* keratitis and to discuss their clinical significance.

## 2. Case

A 42‐year‐old man presented with blurred vision and pain in the right eye. He had a history of atopic dermatitis and open‐angle glaucoma, for which he used brinzolamide and tafluprost/timolol eye drops twice and once daily, respectively. The patient had developed right eye pain 3 days prior and consulted a local ophthalmologist, who diagnosed a corneal ulcer. He was prescribed 0.3% ofloxacin and 0.1% fluorometholone eye drops and was referred to our hospital for further evaluation. He reported using disposable soft contact lenses two to three times per week, always within the recommended 2‐week replacement schedule. He used a multipurpose solution daily to scrub, disinfect, and store the lenses.

**Figure 1:**
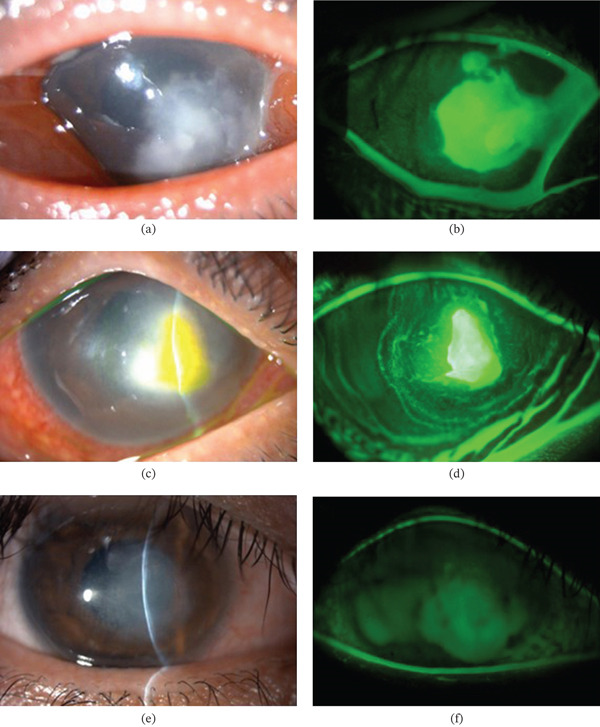
Anterior segment photographs of the patient with *Mycobacterium chelonae* keratitis. (a) Photograph of the anterior segment at initial presentation showing marked conjunctival hyperemia, hypopyon, and a corneal abscess. (b) Fluorescein staining at initial presentation showing a broad corneal epithelial defect. (c) Photograph after 2 weeks showing slightly reduced corneal infiltration and decreased conjunctival edema. (d) Fluorescein staining after 2 weeks showing a slightly smaller epithelial defect. (e) Photograph after 4 months showing reduced cell infiltration and residual corneal opacity. (f) Fluorescein staining after 4 months showing complete resolution of the epithelial defect.

**Figure 2:**
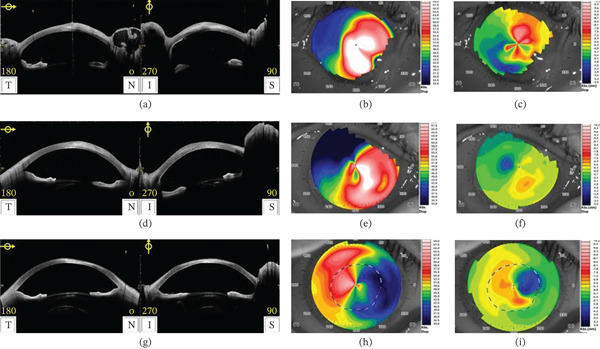
Corneal tomography and topography of the anterior and posterior cornea in the patient with *Mycobacterium chelonae* keratitis. (a) Anterior segment optical coherence tomography (AS‐OCT) image at initial presentation showing corneal infiltration in the nasal area. (b) Anterior corneal power at initial presentation showing corneal thickening and increased anterior power. (c) Posterior corneal power at initial presentation showing increased posterior elevation. (d) AS‐OCT image after 2 weeks showing reduced corneal densitometry. (e) Anterior corneal power after 2 weeks showing persistent elevation. (f) Posterior corneal power after 2 weeks showing partial reduction. (g) AS‐OCT image after 4 months showing resolution of edema and a thinned cornea. (h) Anterior corneal power after 4 months showing reduction in the affected area. (i) Posterior corneal power after 4 months showing decreased values in the affected area.

At presentation, marked hyperemia, hypopyon, anterior chamber inflammation, and conjunctival edema were observed (Figure 1a). The corneal surface exhibited melting, and an epithelial defect extended from the center to the nasal side of the cornea (Figure 1b). The lesion was irregular and consisted of multiple connected satellite lesions. AS‐OCT revealed corneal edema, cloudiness, and hypopyon (Figure 2a); no endothelial plaque was detected. The central corneal thickness was increased to 988 *μ*m. Corneal tomography showed significant edema in the nasal and central anterior cornea (Figure 2b), with corresponding posterior edema (Figure 2c). Best‐corrected visual acuity in the right eye (OD) was counting fingers at 10 cm, and in the left eye (OS), it was 0.06 (1.2 × *S* − 5.75 *D*). Intraocular pressure was 26.5 mmHg in the right eye (OD) and 10.5 mmHg in the left eye (OS). Owing to the irregular lesion shape, presence of satellite lesions with corneal melting, and poor response to the previously prescribed topical antibiotic, the causative organism was unclear. Smears and cultures were performed, and antimicrobial therapy was adjusted based on the results.

Topical steroids were discontinued, as fluorometholone may have exacerbated the infection. Treatment was initiated with 1.5% levofloxacin and 0.3% tobramycin, both eight times daily, and 0.3% ofloxacin ointment once daily. Oral acetazolamide 500 mg was prescribed, and glaucoma eye drops were temporarily stopped to avoid interfering with epithelial healing.

By the fifth day of treatment, the infection showed minimal response. Given the patient′s history of contact lens use and the slow therapeutic response, atypical pathogens were suspected. After 2 weeks, the corneal epithelial defect and cellular infiltration had slightly decreased (Figure 1c,d). AS‐OCT showed reduced corneal edema, with central corneal thickness decreased to 808 *μ*m (Figure 2d), and improvement in both the anterior (Figure 2e) and posterior cornea (Figure 2f).

Corneal scraping cultures showed no bacterial growth, and smears were negative for bacteria and fungi. However, culture from the contact lens case revealed *Delftia acidovorans*, *Stenotrophomonas maltophilia*, *Elizabethkingia miricola*, and gram‐positive bacilli. Based on the AS‐OCT findings, a nonfungal pathogen was suspected. Further microbiological analysis identified nontuberculous mycobacteria, *M. chelonae*, in the contact lens case. Minimum inhibitory concentration values for *M. chelonae* are listed in Table 1. As tobramycin was effective, the same topical antibiotics were continued.

**Table 1:** Minimum inhibitory concentrations of detected pathogens.

Minimum inhibitory concentrations				
Antibiotics	*Delftia acidovorans*	*Stenotrophomonas maltophilia*	*Elizabethkingia miricola*	*Mycobacterium chelonae*
Cefepime (*μ*g/mL)	16	> 16	> 16	—
Amikacin (*μ*g/mL)	> 32	> 32	≤ 8	16
Meropenem (*μ*g/mL)	≤ 1	> 8	> 8	> 64
Sulfamethoxazole and Trimethoprim (*μ*g/mL)	—	—	—	> 8
Tobramycin (*μ*g/mL)	—	—	—	2
Gentamicin (*μ*g/mL)	> 8	> 8	≤ 2	—
Clarithromycin (*μ*g/mL)	—	—	—	0.5
Levofloxacin (*μ*g/mL)	< 0.5	< 0.5	1.0	8
Azithromycin (*μ*g/mL)	8	> 16	> 16	8
Moxifloxacin (*μ*g/mL)	—	—	—	4
Doxycycline (*μ*g/mL)	—	—	—	> 16
Imipenem (*μ*g/mL)	—	—	—	32
Linezolid (*μ*g/mL)	—	—	—	8
Faropenem (*μ*g/mL)	—	—	—	> 64

*Note:* Tobramycin susceptibility for *Stenotrophomonas maltophilia* was not determined. However, this organism is generally considered intrinsically resistant to aminoglycosides, including tobramycin.

As intraocular pressure decreased, oral acetazolamide was gradually tapered. After 2 months, topical levofloxacin and tobramycin were reduced to six times daily. By 3 months, the corneal epithelial defect had fully healed with scarring (Figure 1e,f). Antimicrobial drops were reduced to four times daily, and 0.1% fluorometholone eye drops were reintroduced twice daily.

Following the initiation of fluorometholone, corneal and anterior chamber clarity gradually improved. AS‐OCT showed a decrease in central corneal thickness to 493 *μ*m, within the normal range (Figure 2g). Corneal tomography revealed that the scarred region had become thinner and flatter than in earlier images (Figure 2h). However, irregularities remained in the anterior and posterior corneal surfaces, particularly in the affected area. These asymmetries were difficult to correct with glasses. The posterior corneal surface in the infected area appeared flattened (Figure 2i).

After 4 months of treatment, visual acuity in the right eye (OD) was 0.04 (0.5 × *S* − 4.50 *D*) without the use of rigid gas‐permeable (RGP) lenses; however, best‐corrected visual acuity with an RGP lens was limited to 0.3.

## 3. Discussion

When initial corneal scraping microscopy and culture fail to identify the causative organism of infectious keratitis, culturing the contact lens case can provide valuable evidence for identifying the etiological agent [8]. In the present case, we detected *Delftia acidovorans*, *Stenotrophomonas maltophilia*, *Elizabethkingia miricola*, and gram‐positive bacilli in an ocular surface specimen, and *M. chelonae* was detected in the contact lens case. Clinically, the first four organisms are usually highly susceptible to antimicrobial agents, and if they are the causative pathogens, rapid clinical improvement is expected. However, in the present case, improvement was very slow despite antimicrobial instillation; therefore, we suspected an atypical keratitis caused by *M. chelonae* rather than typical bacterial keratitis. Microbiological diagnosis is crucial but often difficult; therefore, repeated corneal scrapings and specialized culture techniques may be required. In this case, it took 2 weeks to obtain *M. chelonae* results from the contact lens case. With the increasing number of contact lens users alongside rising myopia prevalence [9], the incidence of *M. chelonae* keratitis in contact lens wearers underscores the importance of considering this pathogen in the differential diagnosis of atypical corneal infections. Furthermore, culture testing of contact lens cases is associated with visual prognosis in infectious keratitis [10].

To further support this interpretation, we reviewed the antimicrobial susceptibility profiles. *Stenotrophomonas maltophilia* demonstrated high susceptibility to levofloxacin (MIC < 0.5 * μ*g/mL); however, the patient showed poor clinical response to levofloxacin therapy, suggesting that this organism was unlikely to be the primary pathogen. In contrast, *M. chelonae* exhibited resistance to levofloxacin (MIC 8 *μ*g/mL), which was consistent with the clinical course. Furthermore, the infection responded favorably to tobramycin treatment, which is known to be effective against *M. chelonae*. These findings collectively support that *M. chelonae* was the principal causative organism in this case.

In the present case, the initial use of topical corticosteroids before identifying the causative organism might have suppressed the local immune response and delayed the appropriate diagnosis, which could have contributed to the prolonged course of infection. A limitation of this report is that the causative pathogen could not be directly isolated from corneal scraping specimens. In addition, prior empirical treatment with topical fluorometholone and ofloxacin before presentation may have reduced the microbial burden within the cornea and contributed to the negative corneal scraping culture results.

A comparison with other nontuberculous mycobacterial species, such as *M. abscessus* and *M. fortuitum*, suggests that endothelial irregularities and posterior surface flattening can occasionally be observed in *M. abscessus* keratitis, whereas *M. fortuitum* tends to cause more superficial stromal infiltrates. These findings indicate that AS‐OCT evaluation of the posterior corneal surface may assist in distinguishing *M. chelonae* keratitis from other species of nontuberculous mycobacteria.

Nontuberculous mycobacterial keratitis presents a diagnostic challenge because of its slow progression and atypical features [11]. The clinical manifestations of *M. chelonae* keratitis include conjunctival hyperemia, corneal edema, granular opacity, multiple infiltrates, anterior chamber inflammation, epithelial defects, posterior corneal deposits, and hypopyon, leading to diverse presentations. *Mycobacterium chelonae* keratitis is a rare but serious infection, particularly among contact lens users. Our case highlights the diagnostic difficulties associated with this condition, as the ulcer margins were irregular. This presentation often mimics fungal keratitis, resulting in possible misdiagnosis and treatment delays. Characteristic features include multifocal stromal infiltrates and a “cracked windshield” appearance, which can be easily overlooked and was not observed in our case [12, 13].

The pathogenesis of *M. chelonae* keratitis in contact lens users involves a complex interplay between microbial virulence and host factors. Contact lens wear disrupts the corneal epithelial barrier and alters the ocular surface microenvironment, facilitating bacterial adhesion. *Mycobacterium chelonae* possesses several virulence factors, including biofilm formation and resistance to phagocytosis, which complicate eradication [14]. Recent research suggests that alterations in the ocular surface microbiome due to contact lens wear may promote *M. chelonae* colonization, underscoring the importance of maintaining corneal health in contact lens users [15, 16].

Treatment of *M. chelonae* keratitis is challenging due to its intrinsic resistance to many conventional antibiotics, and no standardized therapy exists [4]. Among ophthalmic topical antibiotics, the minimum inhibitory concentration of levofloxacin against *M. chelonae* was relatively high (8 *μ*g/mL), whereas that of tobramycin was lower (2 *μ*g/mL). Based on these susceptibility results and the limited response to fluoroquinolone therapy, topical treatment centered on tobramycin was continued, which correlated with subsequent clinical improvement. Current literature recommends combination therapy with aminoglycosides, macrolides, and fluoroquinolones to improve prognosis [11]. However, clinical responses do not always align with in vitro susceptibility results. In our case, aggressive topical antibiotic instillation was initiated, and therapy proved effective based on antimicrobial testing. Surgical interventions, such as therapeutic keratoplasty, may be required in cases refractory to medical therapy [11]. Postoperative management demands close monitoring for recurrence and graft rejection, as well as cautious steroid use.

AS‐OCT offers a quantitative and objective assessment of inflammatory and structural changes in infectious keratitis. Despite successful infection control, best‐corrected visual acuity with an RGP lens remained limited in this case. Residual anterior and posterior corneal structural changes observed on AS‐OCT may have contributed to the limited visual outcome, suggesting that optical correction alone may not fully restore visual function after severe *M. chelonae* keratitis. Serial measurement of corneal thickness, stromal reflectivity, and endothelial surface irregularity allows clinicians to monitor the resolution of inflammation and the remodeling process during treatment. These imaging findings can support decisions regarding the timing of corticosteroid reintroduction and help predict visual recovery after infection.

Prevention of *M. chelonae* keratitis requires improved contact lens care and patient education. In particular, it is important to thoroughly wash the contact lens case. Future development of contact lens materials and solutions with enhanced antimicrobial properties may help reduce infection risk. Regular ophthalmic examinations can facilitate early detection of corneal abnormalities in contact lens wearers. Understanding the role of the ocular surface microbiome may also lead to innovative probiotic‐based preventive strategies. A multifaceted approach combining improved diagnostics, preventive measures, and novel therapeutics is essential for managing this challenging infection.

In conclusion, we encountered a patient who developed refractory keratitis due to *M. chelonae* associated with soft contact lens use. Nontuberculous mycobacteria should be considered in treatment‐resistant keratitis, particularly among contact lens users. Furthermore, recognizing topographic changes is important when evaluating visual function after keratitis.

## Author Contributions

All authors attest that they meet the current ICMJE criteria for authorship.

## Funding

No funding was received for this manuscript.

## Ethics Statement

This report was conducted in accordance with the principles of the Declaration of Helsinki. This study was conducted in the Department of Ophthalmology of the University of Tokyo Hospital and approved by the Institutional Review Board of the University of Tokyo Hospital (Examination No. 2217).

## Consent

Written informed consent was obtained from the patient before drafting this case report.

## Conflicts of Interest

The authors declare no conflicts of interest.

## Data Availability

The data supporting the findings in this report are available from the corresponding author upon request. The data are not publicly available because they contain information that may compromise the privacy of the patient.
